# Quality of Life and the Digital Service Landscape: The Moderating Role of Customer Complaining Effort

**DOI:** 10.3390/bs13050375

**Published:** 2023-05-03

**Authors:** Denisa Cristina-Alina Berceanu, Georgeta Pânișoară, Alexandru-Filip Popovici, Cristina Marina Ghiță

**Affiliations:** 1 Department of Applied Psychology, University of Bucharest, 90 Panduri Street, 050663 Bucharest, Romania; denisa.cristina.berceanu@drd.unibuc.ro; 2 Teacher Training Department, University of Bucharest, 90 Panduri Street, 050663 Bucharest, Romania

**Keywords:** digital behavior, digital interactions, customer complaining effort, shopping well-being, quality of life, warranty management applications

## Abstract

The last decade, and more specifically the COVID-19 pandemic, has created a favorable environment for digitalization, which has become a necessary condition in the context of how everyday life is conducted. Even if digital communication and services have become a trend and help brand–customer relationships, brands still have more gaps to close. The purpose of this study was to investigate how consumers’ behaviors and digital interactions impact their shopping well-being and quality of life, and how the level of customer complaining effort affects the relationship between digital behavior and quality of life. This research provides practical implications for companies and marketers that offer digital services and technologies, helping them design and deliver more effective and customer-centric digital experiences. Additionally, it contributes to the growing interest in how digital services and technologies can improve consumer experiences and quality of life. This study surveyed 331 respondents in Romania. Results show that digital behavior influences consumers’ shopping well-being and comes with insights that strengthen the importance of reducing consumers’ cognitive and procedural effort in order to increase their quality of life. The paper discusses the implications for brands that must design easy experiences to gain more loyal customers, the study’s implications and novelty for the warranty area.

## 1. Introduction

The experience of buying products can contribute to an individual’s overall well-being through fulfilling several human needs, such as the need for autonomy or the need to relate to others [1]. Shopping well-being depends very much on the experience the consumer has had while shopping. It varies depending on what the customer is offered and can have an influence on post-purchase behavior in that it encourages loyalty and contributes to the satisfaction the customer has with what they have purchased [2]. Thus, the customer who perceives that the product or the center from which they purchased the product contributes in a positive way to their quality of life will have a high interest in that brand [2]. This is especially important for companies because a high level of customer satisfaction means attracting more customers [3,4].

Even though recent research shows that, in general, the experience of buying products has the potential to contribute to well-being and quality of life [5], there is not much data on what happens beyond this or how customers manage product warranty issues. At this level, problems can arise in the way companies respond to customer complaints about a product they have bought that does not meet their expectations. The way customers react to such contexts varies and can range from negative comments left on the brand’s official page to deciding not to take any action because the process of handling customer complaints is time-consuming and resource-consuming for customers [6]. However, in the long term, the manner in which companies deal with such issues is important, and it is necessary to develop applications or platforms that encourage openness and facilitate interaction with the consumer [7].

Despite the seemingly significant impact of the shopping experience on people’s lives, no research, to our knowledge, has yet considered the impact that customer complaining effort has on the relationship between the decision to use online warranty applications and quality of life.

The findings of this research emphasize the need for companies to prioritize their customers’ well-being. They provide new perspectives on the warranty sector, which has typically focused on technical aspects rather than warranty management and its impact on quality of life. Given that extended warranties come with additional costs for consumers and can impact a company’s revenue, it is crucial to consider other factors that can positively affect brand image. Extended warranties provide more attention to customers, protecting them from product failures and maintenance costs, which in turn improves customer loyalty and enhances brand equity [8].

More than that, the present study provides new insights into this understudied domain. It brings new findings connecting consumers’ online behavior and digital orientation with their shopping well-being. It also shows that not only the shopper’s well-being is influenced by digital services, but also their overall quality of life. Moreover, it addresses the question of how digital services and technologies can enhance consumer experiences and improve overall quality of life, which is a topic that has gained increasing attention.

The impact of customer complaining behavior on consumer outcomes has also been widely studied, but this research adds a unique perspective through examining how the level of effort a customer puts into complaining affects the relationship between their openness to digital services and quality of life. This study has important practical implications for companies and marketers that offer digital services, as it can help them identify the key drivers of shopping well-being and quality of life in the digital era and design more customer-centric digital experiences. Furthermore, the ease or difficulty of the complaining process can either enhance or detract from the impact of digital adoption on consumers’ quality of life. Brands that make it challenging for customers to resolve complaints will ultimately lower their quality of life.

This present study’s results can also be useful for companies and service providers looking to improve customer satisfaction and loyalty through better understanding how to address complaints and concerns related to digital services. Finally, this study can help identify potential barriers to the adoption of digital services and highlight areas where improvements can be made to enhance the customer experience.

This study is presented as follows: In Section 2, we review the literature related to customer complaining effort, shopping well-being, quality of life and use of warranty applications in addition to the conceptual model and hypotheses. Section 3 describes the materials and methods used. Section 4 presents the results of the research, and Section 5 and Section 6 summarize our research contributions, limitations, conclusions and future directions.

## 2. Theoretical Background

### 2.1. Customer Complaining Effort

Consumer complaint behavior has received a lot of attention from researchers, with many studies conducted concerning this behavior. Most studies have focused on the relationship between customer complaint behavior and variables such as age, education, gender and income, with results showing that these variables influence this behavior to some extent [9]. However, few studies have explored other variables, such as the role that quality of life plays.

It is important that consumers’ needs are understood and their expectations met. Effective handling of customer complaints can benefit the company/organization for reasons such as avoiding loss of customers and the identification of product issues and consumer satisfaction information. Customers are usually satisfied if their experience with a product exceeds their expectations of the product. If the product does not meet expectations, then the customer experiences dissatisfaction [2].

Research on consumer complaints is necessary because it provides a clearer picture of customer satisfaction. Complaints show customers’ dissatisfaction with a product and their frustration that their expectations for a product have not been met [10].

Customer complaining refers to the act of communicating a negative or unsatisfactory aspect about a product or service [11]. Complaints are behaviors that are made orally or in writing following incidents that are negatively evaluated by the consumer, motivated by frustration and a desire for compensation for damages [12]. Customer complaining effort refers to the action required of customers to solve their problems [6].

The most common behavior that dissatisfied consumers resort to is negative word of mouth, which has great potential to affect the way a company is viewed. At the same time, it leaves no opportunity to correct or respond to complaints [10]. Dissatisfied customers may leave negative reviews in the online environment. This can seriously affect companies and challenges them to reduce customer complaining effort [6]. Social media messages about products and services can affect consumers’ perceived risk and intention to buy the product or service [13]. People who use social media to follow brands, promotions and retailers are more likely to trust and believe product-related messages, leading to higher acceptance of peer recommendations [13]. On the other hand, although direct complaints allow companies to fix the fault, customers rarely address complaints directly if doing so requires significant effort and energy [6]. This effort may be physical, cognitive or emotional [14,15].

Sales managers should use product or service failures as a chance to enhance customer satisfaction and foster loyalty through implementing consistent standards for that service, even if the organization cannot always prevent the failure from occurring [16,17]. Effective handling of complaints can result in improved customer satisfaction, retention of customers and positive feedback [17,18]. 

In general, this dissatisfaction-related behavior of expressing complaints to companies is a major source of innovative service ideas [19]. In addition, complaints provide one of the main and most direct opportunities to communicate with customers who are dissatisfied with their experiences [20]. Effort is defined in terms of consumer perceptions. In the case of the impact of the supplier’s effort on customer satisfaction, a mediator role is assumed for the perceived quality. In the case of consumer effort, the perceived value is assumed to be a primary mediator. In general, effort is the amount of energy or force put into a behavior or series of behaviors, while perceived effort is the amount of energy that an observer believes an actor has invested in a behavior. Regarding the consequences of such perceptions of effort, research has shown that that there is a positive link between the perceived effort required from the supplier and the consumer’s perception of quality [5].

In the sales environment, managing a complaint involves not only a quick response but also an effort made by the dissatisfied customer. Consumer behavior often reflects disengagement from a poor service experience if the complaint resolution process requires significant effort and energy [17,21]. Customers often choose not to report complaints when they receive poor-quality service. If they perceive the process of complaining as demanding, they may choose to change service providers or use social media to express their dissatisfaction [17,22].

There are studies that have tried to classify consumer complaint behavior into different categories. In one study [15], three motivations that enable consumers to complain were identified: seeking compensation, complaint and personal boycott. In another study [23], the authors proposed the classification of customer behaviors into three distinct actions: voice, which refers to complaints made directly to the service provider; spreading negative reviews on social media; and complaining to outside organizations.

Customers who complain directly to the company about unsatisfactory service offer service providers an opportunity to fix the problem, but this comes at the cost of the customer’s time and effort [3,17]. Research indicates that when the effort required is significant, customers often opt not to pursue a complaint and instead share negative reviews [24]. To address this, managers should implement strategies that reduce the effort required of customers through providing quick and accurate solutions. In the following paragraphs, we will briefly present three factors that influence this effort in consumer complaining behavior: time, cognitive effort and affective effort.

Individuals may view time management differently depending on the situation, viewing it as either an investment or a cost. Time is seen as a finite resource that must be allocated. This has also been highlighted by the way in which it influences consumer satisfaction, service comfort and quality perceptions [25]. When consumers complain about a poor service experience, the earlier the remedy, the more likely consumers are to regain their satisfaction. Studies indicate that preprocessing expectations are considered more frustrating than in-process expectations, because while waiting for preprocessing, clients do not know how soon services will be initiated, which can cause them anxiety [26].

Cognitive effort is another energy resource that individuals use to process information. It is at the heart of decision-making and consumer research [27]. In consumers’ behavior, they seek to preserve their cognitive effort when interacting with service providers [26]. Cognitive effort involves multiple cognitive functions and operations, such as appreciation, classification and reasoning [10].

Affect is an emotion that a person experiences on a subjective level, usually triggered by what is happening in the environment [17,28,29]. This is important because affect can determine how a potential buyer evaluates products and decides whether to buy them [30]. Affect also plays a role in the buyer’s perception of the effort they put into making a complaint. Thus, positive affect is associated with a perception of less effort, while negative affect will also imply a perception of more effort [31].

Consumer effort refers to the physical, mental and financial resources the consumer uses to obtain a product [3,32]. This effort may consist of financial expenditure and time invested but also perceptions, memories and judgements on the basis of which a decision to buy or not to buy will be made [33,34,35,36]. Consumer effort is usually defined in terms of consumer perceptions. Research on the effects of individual effort, conducted outside the context of consumption, reveals a model of ambiguity regarding the impact of effort on evaluations. Dissatisfied customers may not complain but may leave the company’s services due to company mistakes, billing errors and service disasters. Moreover, others may leave because of an unfavorable encounter with the service, such as careless, rude or unresponsive employees without knowledge. If customer complaints are not handled properly, the negative consequences can be far-reaching. Dissatisfied customers will not only interrupt their patronage, but are also likely to spread a negative message, endangering the company’s image [37,38,39]. 

Customer complaining behavior refers to responses triggered by perceived dissatisfaction that is neither psychologically accepted nor quickly forgotten in the consumption of a product or service. Customer complaint behavior is a complex phenomenon that is reflected in the number of alternative definitions proposed to explain this type of behavior [40]. Traditionally, the common determinant of complaint behavior has been described as dissatisfaction due to inadequacies of integrity, reliability, responsiveness, availability and functionality [40]. Therefore, consumer dissatisfaction is the result of the discrepancy between expected and achieved performance [37]. Dissatisfaction is based on non-confirmation of expectations [41] and is a customer experience that is lower than perceived expectations. According to this [40], customer complaint behavior is a function of dissatisfaction.

Other authors [42,43] have stated that dissatisfaction is a significant feature that is attributed to complaints. Moreover, dissatisfaction can be caused by a lack of confirmation of purchase expectations which lead to a legitimate complaint behavior [44]. Many studies of consumer satisfaction and dissatisfaction have used the paradigm of disconfirmation [41].

### 2.2. Shopping Well-Being

The concept of shopping well-being can be viewed and analyzed through appealing to a concept in the literature, namely, consumer satisfaction with various areas of life throughout the consumption process, from purchase to consumption to disposal of goods and services [45]. It is important to note the difference between shopping wellness and overall consumer wellness. Well-being during shopping refers to the psycho-emotional balance of the individual and their life satisfaction, and the well-being of consumers refers to the effects of the different experiences that individuals have over time on the activities of purchasing products, coming in direct connection with the actual purchase action [46].

The buyer profile includes a set of actions regarding the purchase of certain goods and services to meet their personal and family needs. In other words, shopping is essential for achieving and meeting more complex needs. An individual can go to the supermarket every day to prepare meals for his family. This is the utilitarian function of shopping, and much research has documented that the satisfaction of needs has effects on this activity. For example, the level at which stores contribute to customer satisfaction and perceived quality of life is based in part on the functional aspects of stores and other outlets in providing desired goods and services at acceptable levels [47]. However, it is important to note that shopping goes beyond its utilitarian value, having also a hedonic and affective character. This is based on the fact that the experience of shopping provokes positive emotions in the customer’s relationship with the retailer [48].

Regarding the analysis of the strong impact of shopping on people’s well-being, there are not enough studies that address the situation of how shopping influences the general feeling of well-being. One study [49] showed that shopping can contribute to well-being, but much of the available research focuses on the detrimental effects of compulsive shopping on quality of life. Shopping well-being pertains to the positive effects of shopping experiences on quality of life, while compulsive shopping examines the negative consequences [49].

Well-being in shopping is also defined by the impact perceived by a buyer on their satisfaction in important areas of life. This concept refers to the emotional condition and degree of contentment that shoppers can attain based on their shopping experiences.

Within the subdomain of experiences related to the purchase of products are emotional experiences related to stores. The shopping experience covers a wide range of experiences, such as the purchase of consumer goods and services, socializing with retail staff and other buyers and entertainment. These experiences are multiple and play an important role in the overall quality of life [50,51].

Shopping contributes to life satisfaction through ensuring that consumers’ needs are met by the products they purchase and also provides a space where shoppers can socialize with others. Thus, stores can make a significant contribution to community well-being through providing a meeting place for individuals to come together and experience a sense of community. The development of well-being in the field of consumer psychology presents imprints on a social level and on a personal level, as well as by spending free time, serving to increase general satisfaction with life [51].

Some research has highlighted the relationship between the level of abandonment of a particular product by the consumer and well-being [45]. In the analysis of this profile, consumers consider the shopping experience beneficial and a means to achieve physical and mental balance, which, in turn, contributes to shopping satisfaction and the overall quality of life of consumers [49].

Convenience plays an important role in installing the well-being of shopping through training and developing all four elements of shopping well-being: the consumer, the social environment, fun and socialization. An inconvenient store leads to difficulties for buyers, leading to the avoidance of planned purchases of goods and services. A store that is inconvenient makes it difficult for shoppers to socialize with others and experience well-being. In order for a consumer to feel a sense of well-being during their purchasing experience, it is crucial that they are content with the services provided. This can, in turn, have a substantial impact on their overall life satisfaction [49].

When consumers perceive a store as having a high level of orientation towards them, this leads to the development of their well-being in their free time. Shops can achieve this through providing various entertainment facilities and food and beverage facilities for children and adults. In addition, entertainment facilities not only increase well-being in leisure time, but also social well-being. People meet in big stores to socialize, to consume entertainment services (for example, to watch a movie), to have lunch or dinner, to have ice cream or to discuss community events. Shoppers perceive stores differently. Factors related to oneself lead to perceptions aimed at increasing well-being. Shoppers like to shop in stores where they meet people they can identify with. This process of matching is called “congruence of self-image” [52].

Another important feature is the sense of personal expressiveness, which again relates to well-being. Those consumers who feel this at a store, in the sense that they identify themselves with what is made available to them, have a higher level of well-being than those who do not find something that reflects their own identity [46,53].

Consumers with a high level of well-being are prone to be organized, demanding, disciplined, diligent, reliable, methodical and intentional. Thus, they are more prone than consumers with a low level of well-being, who avoid fulfilling their responsibilities thoroughly and correctly, to take the initiative in solving problems, to remain committed to the performance of work, to respect policies and to remain focused. Recent research has shown that store managers perceive personal satisfaction and well-being as the most important attributes related to consumer receptivity [54].

Therefore, a consumer’s purchasing decisions are considerably influenced by several personal characteristics. On the other hand, when discussing such a purchase, consumers’ perception of a certain brand must also be considered, as studies show that people tend to equate their needs more with certain brands of products and the level in which these needs lead to the improvement of well-being [55]. This may be because people prefer products that they perceive as having a personality similar to their own. Therefore, apart from each individual personality trait, the whole concept of personality impacts the buyer’s behavior, and implicitly their well-being, during shopping. An analysis of the consumer profile in the current context is particularly important because it encompasses the requirements and preferences of customers and contributes to the development of the process of personal satisfaction. Through the purchase decision, consumers influence the sales and even the profile of a company and, therefore, any marketing activity must be analyzed and carried out in relation to the needs of consumers.

Dissatisfaction is a significant feature that is attributed to complaints and can be caused by the lack of confirmation of purchase expectations, which leads to a legitimate complaint behavior [42,44]. Many studies on consumer satisfaction and dissatisfaction have used the paradigm of disconfirmation [41].

### 2.3. Quality of Life

Concerning quality of life, recent research shows that the shopping experience can contribute to well-being [5,46,56]. Consumer well-being is high when customers are satisfied with the experiences of purchasing products and using them. This has an important role in life satisfaction, as marketplace experiences reflect in the overall satisfaction with life [57]. Additionally, for different brands and companies, the experience that customers have when they buy is relevant because if the experience is a satisfying one, then customer loyalty and positive word-of-mouth communications increase [46].

In general, the experience that customers have when buying a product and the experience with the product itself reflect on the wider life domain of the shopper [45]. Since previous research on this concept is quite scarce and the conceptualization of consumer well-being has primarily focused on retail possessions and institutions, it has been proposed to look at this concept another way, which is much broader and looks at the whole consumption process [45]. This conceptualization refers to five major dimensions of satisfaction with consumer goods and services, namely: acquisition, possession, consumption, maintenance and disposition; it is based on the “bottom-up spillover theory of life satisfaction,” which assumes that overall satisfaction with one’s life is directly related to satisfaction with all domains and sub-domains of life [45]. Thus, the greater the satisfaction with areas of one’s life such as health, family, work or leisure, the greater the satisfaction with life in general [45]. In turn, satisfaction with a specific area of life such as the area of consumer life is directly influenced by satisfaction with certain things (events and concerns) that have happened within that area [45]. According to the authors, acquisition satisfaction is determined by the shopping experience, which can be satisfying or less satisfying [45]. Possession satisfaction refers to the satisfaction that results from owning consumer goods. Related to this is consumption satisfaction, which refers to the satisfaction that comes from using products and services. The difference between the two is that possession satisfaction focuses on the positive aspects that arise from owning a product, whereas consumption satisfaction comes from using the product or service.

Maintenance satisfaction is the satisfaction that consumers feel and experience when they seek to repair or service a purchased product, and it comprises two dimensions, namely, satisfaction with maintenance and repairs provided by those who provide such services as well as satisfaction with services that facilitate maintenance and repairs by the owners themselves [45]. Disposition satisfaction looks at the degree of satisfaction consumers feel when they are in a context that involves the disposal of the product they own and the environmental friendliness of the product at the time of its disposal.

### 2.4. Use of Warranty Applications

Making a complaint directly about a non-conforming product or service is not a simple task and few consumers dare to do it. For someone to initiate a complaint, the level of dissatisfaction must be high, the problem must be consistent and the customer must believe that there is a solution to resolve it [58]. Companies would prefer customers to complain to them so that they can start the procedures to respond to customers and resolve the problem. Because of the many complaints that customers make online, many firms employ people to monitor social media platforms and profile sites and respond to complaints and referrals that customers make [58]. In this way, sales organizations demonstrate that they care about the customer experience, but at the same time move the conversation into a private space to limit the image and reputation damage caused by negative word of mouth [58]. For companies, it is essential to have the ability to see through the eyes of their customers and to develop and deliver successful services [17].

When customers have an unpleasant experience, they will evaluate the experience and their ability to deal with it. At the same time, they will assess the degree to which it violated their expectations and react accordingly [22]. In this sense, many reactions can accompany this experience, starting with general satisfaction regarding the service/product purchased and potentially reaching frustration, anger, disgust, contempt and other emotional reactions that influence consumer behavior [22].

However, research in this area shows that the way customer complaints are handled is not always satisfactory. Although the interaction takes place in a private space, in another study [59], it emerged that many firms choose to respond with a series of predefined phrases based on an intelligent chatbot, which is insufficient to resolve customer complaints as they have certain peculiarities that cannot be addressed similarly.

Thus, for firms/companies, an application that manages client complaints would ease the way they interact with clients and at the same time would facilitate this process and serve the interests of the firm but also ease the customer experience. For example, in the case of having an online complaints system, one advantage already noted is that it is a cost-effective option and is more efficient in handling complaints than traditional customer service [19,60].

**Figure 1:**
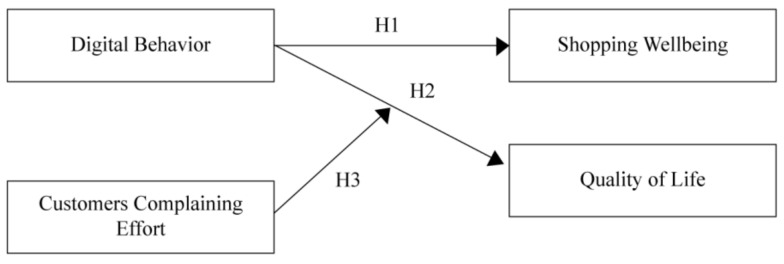
Conceptual research model.

Based on the literature review, the following hypotheses were proposed. Figure 1 also presents the conceptual research model based on the proposed hypotheses.

**Hypothesis** **1** **(H1).** *Digital behavior and positive emotions influence shopping well-being*.

**Hypothesis** **2** **(H2).** *Digital behavior and positive emotions influence consumers’ quality of life*.

**Hypothesis** **3** **(H3).** *Customer complaining effort moderates the relationship between digital behavior adoption and quality of life.*

## 3. Materials and Methods

### 3.1. Participants

Data obtained for the current study (March 2022–December 2022) were extracted from the largest project on Romanian consumers’ digital behavior, which is ongoing.

A total number of 331 respondents was used for the current research with a 100% response rate. All the completed questionnaires were valid. The demographic profile of the sample is shown in Table 1.

### 3.2. Survey Procedure

The questionnaires used for this study were built using Google Forms. Participation in the present study was voluntary. The data were collected from 331 consumers from Romania, using two methods: (1) sending the study link via social media or email to specific respondents and (2) using the snowball technique. Demographic data were collected for gender, age, level of education and professional status. All participants were informed about the objective of the study (the fact that the main purpose of the study is to collect data about their perception of online shopping, their relationship with brands and their quality of life), the procedure and instructions of the study; they were also assured about the confidentiality of their data. Participants were also informed that the researchers are interested only in their opinions and that the research data will be used only for the purpose of data analysis, with the reminder that participation in this research does not require their obligation to participate in other future stages. The included participants agreed with the written informed consent form no. 94/08.12.2021 before starting to complete the questionnaires.

### 3.3. Measures

The survey consisted of five sections. The first section was designed to measure the demographic data of the respondents specified before. The second section contained questions developed to measure the consumer profile through both Likert scales and descriptive multiple-choice questions. Questions measuring consumer profile were pretested in a previous stage and then adjusted accordingly after the initial analysis. In this regard, some questions were reworded to enhance comprehension of the subject matter, while for other questions, we changed the answer choices to better align with the research focus. Items used to measure consumer profile can be seen in Appendix A.

- The third section was designed to measure the respondents’ quality of life. Quality of life was measured using the QOLI^®^ questionnaire [61] which consists of 16 life areas, according to the author, divided into 4 main categoriesPrimary needs: health, self-esteem, goals-and-values and money.
- Activities—occupations/hobbies: work, play, learning, creativity and help.
- Relationships: love, friends, children and relatives.
- Environment: home, neighborhood and community.

QOLI^®^ is a measure of life satisfaction, positive psychology and positive mental health [62]. It has been used extensively in well-being outcomes, descriptive and case studies in both clinical and nonclinical settings (i.e., coaching populations) and found to be empirically validated. According to the model, quality of life refers to the subjective evaluation of the degree to which a person has satisfied his needs, achieved his proposed goals and fulfilled his desires [63]. Thus, life satisfaction or quality of life is perceived as the difference between what a person wants and what he actually has. Examples of questions are “How important is love for your happiness?” and “How satisfied are you with the love from your life?”. These types of questions are adapted for all the other life areas. The questionnaire was validated for the Romanian population using Test Central [64]. The internal reliability of QOLI has been demonstrated to be at a very good level, with a Cronbach’s α of 0.82 (m = 48.82, SD = 7.99). 

The fourth section was designed to measure consumers’ shopping well-being. Shopping well-being was adapted after the SHWB questionnaire (belief that shopping contributes to personal and one’s family’s quality of life) [49]. The English version of the Shopping Well-Being Scale was first translated into Romanian and then verified by professional translators to see if the meaning of the English questions had been preserved and would be correctly understood by respondents. Shopping well-being was measured using a Likert scale ranging from 1 (strongly disagree) to 7 (strongly agree), with examples of items such as: “Thinking about shopping, I feel that my shopping contributes significantly to my own personal well-being” or “I feel that my shopping contributes significantly to my family’s quality of life overall”. The internal reliability of the SWLS has been demonstrated to be at a very good level, with a Cronbach’s α of 0.92 (m = 3.73, SD = 1.39). 

The fifth section evaluated customer complaining effort using the Consumer Complaining Effort Scale [65], which consists of four factors as the following: procedural effort, cognitive confusion, time-related effort and affective effort. Consumer complaining effort was measured using a Likert scale ranging from 1 (never) to 5 (always), with examples of items such as: “It took great time and effort to provide documents required to prove my dissatisfaction” (procedural effort), “I did not know which channel I should use to have my problem resolved” (cognitive confusion), “It took me a long time to have my problem resolved” (time-related effort) or “I was angry about this experience” (affective effort). The English version of the Consumer Complaining Effort Scale was first translated into Romanian and then verified by professional translators to see if the meaning of the English questions had been preserved and would be correctly understood by respondents. CFA analysis was performed using IBM SPSS AMOS 26 in order to verify the model fit. To accomplish this, the chi-square (χ2) value and a series of goodness-of-fit indices were examined including the chi-square to the degrees of freedom ratio (i.e., χ2/degrees of freedom [df]), comparative fit index (CFI), Tucker-Lewis Index (TLI), and root mean square error of approximation (RMSEA). The fit indices of the 16-item model are: χ2 = 299.23 (df = 98), χ2/df = 3.05, CFI = 0.95, TLI = 0.94 and RMSEA = 0.079, indicating a good model.

## 4. Results

The discussion of the results is organized into two sections: (1) consumer profile analysis that includes descriptive and preliminary results and (2) hypothesis testing.

### 4.1. Consumer Profile Descriptive and Preliminary Analysis

Consumer profile was analyzed in terms of the respondents’ shopping habits and their attitude towards digital applications. In terms of the consumers’ preferences for online vs. offline shopping, most of the respondents prefer to go directly to physical stores (58%) to purchase their preferred products, while 26% of respondents prefer a mixed way to shop. Only 16% of respondents declared that they prefer to shop 100% online. Regarding the time spent shopping, most of the respondents (42%) spend 1–2 h per week, while 29% spend 2–5 h, with 26% of consumers preferring to finish the shopping process in less than 1 h. The motivation behind online usage is predominantly related to time management (23%), larger stocks found online (18%), comfort (15%) and the perception that online shopping is simpler than going directly to a physical store (15%). In terms of budget, 48% of consumers spend less than EUR 60 during shopping, while 45% spend EUR 60–120 on the products they buy weekly. Regarding the payment methods used daily, 158 (47.7%) respondents pay with a card as the primary method, 78 (23.6%) respondents use mobile applications, 91 (27.5%) respondents prefer to pay with cash and 4 (1.2%) respondents prefer to pay with a meal voucher card. Positive experience with a brand (35%) is the most important aspect of choosing products, with 21% of respondents buying only from their preferred brands. This indicates the importance of brands in the consumers’ lives in general and in the shopping experience in particular.

The data from Table 2 present the means and standard deviations of responses to the Likert-scale questions. We can observe that consumers from our sample have a very positive attitude towards online applications, being open to change from traditional services management to online management. Their view of digital services is predominantly positive, both from the importance of applications in their relationship with brands and from their openness to use any new application their preferred brands could develop. Even if the warranty management system is easy to use for them, customers are totally open to change to digitized solutions. The usage of brand loyalty cards also presents above-average scores, meaning that most of the respondents are brand-loyal and recurrent consumers. Emotional state during shopping seems to not touch higher scores. When it comes to overall shopping well-being and quality of life, customers tend to have a moderate level. The results on the types of customer complaining effort have revealed that the highest levels were recorded for procedural and cognitive effort. Nonetheless, all four types of complaining effort appear to exhibit moderate-to-high levels. Regarding the relationship between consumers’ quality of life and shopping well-being, results show that there is no significant correlation between the two concepts (*p* > 0.05).

### 4.2. Hypotheses Testing

The proposed hypotheses were tested through moderation analysis [66] using PROCESS v4.0, regression analysis and Pearson correlation using IBM SPSS Statistics 20. In the moderation analysis, we controlled for the impact of covariates (i.e., gender, age) to gain alternative explanations for our results. Customer complaining effort was selected as a moderator due to its significance in the customer journey and the insights it provides into how satisfied or dissatisfied customers are with the services they are using, as highlighted in both the marketing and consumer psychology literature. Through this approach, we wanted to (1) highlight the specific issues consumers experience, (2) identify the pain points related to customer experience to enhance the consumers’ quality of life and (3) provide valuable insights into areas that require improvement in digital services.

#### 4.2.1. Hypothesis 1 (H1). Digital Behavior and Positive Emotions Influence Shopping Well-Being

##### H1. (a) Factors Influencing Consumers’ Shopping Well-Being

Hypothesis 1 states that digital behavior adoption and the positive emotions consumers have while they shop influence their shopping well-being. To test the first hypothesis, Pearson correlation was used. Variables included one’s attitude towards online applications, digital behavior adoption, digital warranty management adoption, the importance of online applications, the usage of brand loyalty cards, one’s emotional state during shopping and the ease of the warranty management process. Results indicate that digital behavior outcomes indeed have a positive influence on the consumers’ shopping well-being, as per the following: online applications attitude (*r =* 0.15, *p* < 0.01), digital behavior adoption (*r* = 0.22, *p* < 0.01), digital warranty management adoption (*r* = 0.15, *p* < 0.01), online applications importance (*r* = 0.19, *p* < 0.01), brand loyalty card usage (*r* = 0.23, *p* < 0.01) and emotional state during shopping (*r* = 0.22, *p* < 0.01). However, results show lower levels of coefficients indicating low correlations between digital habits and positive emotions during shopping, on the one side, and shopping well-being on the other side. Warranty management and ease of use have no significant influences on shopping well-being.

##### H1.(b) Predictors of Shopping Well-Being

Considering the positive correlations presented, linear regression analysis has also been performed to verify the prediction relationship between consumer behavior outcomes and shopping well-being. Same variables were included in the analysis. Gender and age were introduced as covariates. Results have shown that only emotional state during shopping (*β* = 0.17, *p* < 0.01) and the usage of loyalty cards (*β* = 0.12, *p* < 0.05) were significant predictors of consumer shopping well-being. In other words, the percentage of variation in shopping well-being due the consumer’s emotional state during shopping is 17%. Similarly, the usage of loyalty cards explains 12% from consumers’ shopping well-being. Gender and age were not significant predictors of shopping well-being.

#### 4.2.2. Hypothesis 2 (H2). Digital Outcomes Contribute to Improvements in Consumers’ Quality of Life

##### H2. (a) Factors Influencing Consumers’ Quality of Life

Hypothesis 2 states that digital behavior and positive emotions influence consumers’ quality of life. To test the hypothesis, Pearson correlation was used. We included the same consumer behavior variables as previously. Results indicate that almost all the online consumers behaviors and perceptions included in the analysis have a small positive impact on quality of life, as per the following: applications importance (*r* = 0.16, *p* < 0.01), warranty management ease of use (*r* = 0.20, *p* < 0.01), warranty application openness (*r* = 0.15, *p* < 0.01), brands application openness (*r* = 0.22, *p* < 0.01), attitude towards online shopping (*r* = 0.22, *p* < 0.01) and emotional state during shopping (0.13, *p* < 0.05). On the other hand, loyalty card usage has no influence on consumers’ quality of life.

##### H2. (b) Predictors of Quality of Life

Linear regression analysis was also performed to verify the predictive relationship between consumer behavior outcomes and quality of life. The same predictors were included in this analysis. More specifically, we expected that consumers’ habits and behaviors are predictors for the quality of life. In this regard, the hypothesis was partially confirmed.

Results have shown that only ease of warranty management was a significant predictor of quality of life (*β* = 0.15, *p* < 0.05). This indicates that the percentage of variation in quality of life due to the ease of warranty management is 15%. Gender and age were not significant predictors of quality of life.

#### 4.2.3. Hypothesis 3 (H3). Customer Complaining Effort Moderates the Relationship between Openness to Digital Services and Quality of Life

##### H3. (a) Customer Complaining Effort Moderates the Relationship between Digital Services Adoption and Quality of Life

In the first model, we hypothesized that the interaction between consumer complaining effort and the adoption of digital services would relate to a lower quality-of-life score. More specifically, we expected that higher scores of consumers complaining effort would lead to lower scores of quality of life when users are open to digitalized services. We included each consumer complaining factor in a separate moderation analysis. Gender and age were added as covariates. In this regard, the hypothesis was partially confirmed. Only procedural (*β * =  −0.84, ΔR^2^ = 0.08, F = 5.46, SE = 0.34, *p*  <  0.01) and cognitive (*β * =  −0.70, ΔR^2^ = 0.09, F = 6.44, SE = 0.30, *p*  <  0.01) effort presented significant coefficients. Among the controlled variables, none of them were related to quality of life. At higher levels of procedural and cognitive effort, the effect of the adoption of digital services on quality of life decreases.

##### H3. (b) Customer Complaining Effort Moderates the Relationship between the Adoption of Warranty Applications and Quality of Life

For the second moderation model, we hypothesized that the interaction between consumer complaining effort and the adoption of digital warranty management applications would relate to a lower quality of life score. We expected that higher scores of consumers complaining effort would lead to lower scores of quality of life when users are open to digitalized services for warranty management. We again included each consumer complaining factor in separate moderation analysis with gender and age as covariates. In this regard, the hypothesis was partially confirmed. Similar to the above results, only procedural (*β * =  −0.77, ΔR^2^ = 0.06, F = 3.84, SE = 0.33, *p*  <  0.01) and cognitive (*β * =  −0.56, ΔR^2^ = 0.07, F = 4.78, SE = 0.28, *p*  <  0.01) effort presented significant coefficients. Among the controlled variables, none of them were related to quality of life. At higher levels of procedural and cognitive effort, the effect of the digital warranty management adoption on quality of life decreases.

Considering the above results, Hypothesis 3 was partially supported. However, we observed that the results were preserved during the second model, indicating that customer effort has a significant impact on the relationship between digital behavior adoption (no matter the industry where the behavior is applied) and satisfaction with life. The results of the present study are summarized in Table 3.

## 5. Discussion and Conclusions

The last decade, and more specifically the COVID-19 pandemic, created a favorable environment for digitalization, which became a necessary condition in the context of how everyday life is conducted. However, not only is it a significant factor that influences people’s interactions (at work or in their social relations), but it is a necessary tool that helps brands develop meaningful relationships with their customers. Through digital interactions, brands are able to both improve customers’ shopping experience and also communicate with them through social media in order to increase their level of engagement [67].

However, even if digital communication and services have become a trend and have helped brand–customer relationships, brands still have more gaps to close. Over the years, digitalization has come with a higher level of competitiveness and has also presented a challenge for companies to keep up with consumer behavior changes [68]. Since customers have lower attention spans [69] and are kept in their daily routines and burdens, these shifts in their behaviors and emotions make brands more aware of the importance of creating easy processes for them. 

Our results strengthen the idea that companies must be more careful regarding their clients and their well-being. This research also comes with new insights into the warranty area, whereas most existing studies are more focused on technical approaches [70] and not directly on warranty management and quality of life. Since extended warranties for goods come with extra costs for consumers [8] that impact companies’ revenues, it is important to focus more on consumers’ other life factors that could positively impact brand image. As studies have shown, Dell and Apple have declared that extended warranties (EW) revenue accounts for 26% and 43% of their net income, respectively. Because EWs give clients more attention, protecting them from product failures and unquantifiable maintenance costs, this improves consumers’ loyalty and brand equity [71].

First, the present study comes with new insights that link consumers’ online behavior and digital orientation to their shopping well-being. According to our results, digital services are important aspects that brands must consider if they want to improve consumers’ quality of life through shopping. Results are in accordance with studies that show that digitalization in this domain has the potential to contribute to increased well-being through the accessibility it offers [72,73,74].

However, digital adoption is not the only factor that determines shoppers’ well-being. Loyalty program adoption contributes to consumers’ well-being in the shopping experience as well. As past research showed, this can be explained through the fact that when customers experience high brand loyalty and brand–community belongingness, these influence them to use the brand more often. The more they use or frequent a brand, the more likely they experience positive feelings intensely, not just in one life domain, but in several (leisure life, social life, work life, physical/sensual life, etc.) [75,76]. More than that, as our results show, customers tend to be loyal to some brands and are also influenced in the buying process by a positive experience with a brand. In other words, when people are part of loyalty programs, they choose the brands because they experienced positive events in regard to that brand, so these positive feelings are transferred to the well-being they experience in the shopping process.

Second, our research highlights that shoppers’ well-being is influenced not only by digital services but also the consumers’ quality of life. In this regard, past research shows similar results, in which the quality of life is influenced by services that are related to information and communication technologies. The use of these services may have multiple contributions to quality of life because it facilitates access to different services that are considered important for consumers [77].

Since these factors only influence consumers’ quality of life in some ways, none of them can predict it. However, what can be an important predictor of consumer satisfaction with life is the easiness consumers perceive in the management of products or services. As some studies show, this is an important variable that encourages consumers to use such services and increases their trust in a particular brand [77,78,79,80].

Finally, customers’ complaining effort modifies the effects of digital adoption on consumers’ quality of life. No matter the industry where customers apply and use digitalization, when brands make the complaint-solving process difficult (in terms of the procedures used or the cognitive effort implied), this will decrease consumers’ quality of life.

Brands are everywhere. We work with them and buy from them every day. However, it is more than evident that sometimes, some problems, no matter their nature, can interfere with this relationship (they could be some questions we have for the company at the beginning of our relationship or some serious problems they need to solve). When brands make consumers’ lives difficult through the fact that they do not offer clear processes, but instead they come with too much paperwork or cognitive confusion (when consumers do not know when and where to address their questions) in the problem-solving journey, that will affect consumers’ peace of mind and, through this, their overall quality of life. Given that levels of stress in this century have reached high levels [81,82,83,84,85], technology comes to ease people’s lives and increase their peace of mind [85,86,87,88]. However, when brands come with another layer of difficulty in their relationship with the customers that joins their daily pressures and stress, this will also interfere with their overall life. Putting too much effort into understanding procedures or into finding someone that can help you means investing time in a cognitive or procedural burden. As past research shows, customer satisfaction depends on the effectiveness of brands in addressing any issues related to the purchased product. Effective management of this aspect increases customer satisfaction and loyalty with the brand [87].

Since consumers may not give up on the relationship after the first offense, it is important for brands to understand that making consumers’ lives easier will not only increase their level of satisfaction but will improve the relationship between brands and customers. Having a positive impact on customers’ quality of life will make them come back recurrently because of their positive experiences. With peace of mind and a positive mindset, a happy customer will become a loyal customer and, further, a customer that identifies with the brand [89,90].

The findings of this research also have several important managerial implications for companies. Managers should focus on developing user-friendly digital services that are easy to use and require minimal effort. This can include designing interfaces that are intuitive and straightforward, providing clear instructions and support and minimizing the number of steps required to complete tasks. Through providing clear information about the features and functionalities of their services as well as the support and resources available to customers, managers can reduce customer frustration and increase satisfaction. This can help them build trust and loyalty among customers, and ultimately increase the success of their digital services.

Finally, managers should develop metrics and tools to measure the cognitive and procedural efforts required to use digital services and how they impact the quality of life of customers. This can help managers make informed decisions about the design and development of digital services that are customer-centric and focus on improving consumers’ quality of life.

In conclusion, digitalization is not only a trend. It is a necessary solution that makes companies persist in a competitive environment. However, providing simple and easy processes for customers and being more oriented on how they impact their life satisfaction and quality of life must be not only a challenge, but an opportunity to gain customers on their side.

## 6. Limitations and Future Lines of Research

The current research can lay the groundwork for future studies through addressing its limitations and exploring the variables’ relations in other cultures or countries with different behavior patterns.

It is important to note that the study was conducted in the aftermath of the COVID-19 pandemic, so the results regarding quality of life, shopping satisfaction and behaviors may have been impacted by pandemic-related fears and anxieties. Further research should be conducted to validate relationships between concepts from a longitudinal perspective and to compare the findings with those recorded prior to the COVID-19 pandemic, in order to assess the lasting effects of this period on consumers’ mindsets over time.

Additionally, we must take into consideration that the present study consists of Romanian consumers. In this regard, future studies should gather larger samples that can provide reliable results across multiple countries, with a distribution that accurately reflects the European population. This will enable generalizations to be made about Europe as a whole. This line of further research will allow comparative studies to be carried out between the results obtained on Romanian consumers and those from other countries.

Another limitation of this study is that the intention to use online applications in specific areas of social life was not considered. To compare our own results more comprehensively with those of other research, other variables (e.g., different instrument sizes, attitude toward online applications, intention to make an effort, etc.) will be considered in the future. Further, researchers must verify the impact of customer effort on different industries in order to check if the results are different depending on the customer’s journey.

Future research must consider the important link between shopping well-being and quality of life across different industries, for both digital and non-digital customers. Comparative studies focusing on how shopping behavior and preferences change over time, particularly in relation to age, could provide valuable insights into the relationship between shopping and well-being. It would also be important to consider longitudinal studies of individual shopping behaviors and well-being over time in order to understand how these variables interact and change over time.

## Figures and Tables

**Table 1:** Respondents’ demographic characteristics.

	Frequency	N
Gender		
Females	82.2%	272
Males	17.2%	57
Other	0.6%	2
Age		
Less than 20 years	33.8%	112
21–30 years	35.1%	113
31–40 years	17.2%	57
41–50 years	10.6%	35
51–60 years	3.9%	13
More than 60 years	0.3%	1
Education		
Higher education	61.9%	205
Lower education	36.5%	121
Specialty courses	0.6%	2
Professional status		
Students	53.8%	178
Employees	44.4%	147
Unemployed	1.5%	5
Retired	1.2%	4

**Table 2:** Means and standard deviations regarding consumer perceptions and behaviors, quality of life and shopping well-being.

Variable	Mean (SD)
Consumer perceptions and behaviors	
App attitude	5.22 (1.09)
Online applications adoption from brands	4.02 (1.14)
Digitized warranty management adoption	4.00 (1.19)
Warranty management ease of use	3.57 (1.18)
Online applications importance	3.72 (1.65)
Brand loyalty cards usage	3.29 (1.31)
Emotional state during shopping	2.44 (.70)
Shopping Well-being	3.73 (1.39)
Quality of life	48.82 (7.99)
Customer complaining effort	
Procedural Effort	3.46 (1.00)
Cognitive Effort	3.5 (1.18)
Time Effort	3.27 (1.21)
Affective Effort	3.37 (0.78)

**Table 3:** Study results (N = 331).

Hypothesis	Outcome	Variables	Coefficients	Sig. (2-Tailed)
**H1**	Shopping Well-Being	Correlates		
		Online applications attitude	*r* = 0.15 **	<0.01
		Digital behavior adoption	*r* = 0.22 **	<0.01
		Digital warranty management adoption	*r* = 0.15 **	<0.01
		Online applications importance	*r* = 0.19 **	<0.01
		Brand loyalty card usage	*r* = 0.23 **	<0.01
		Emotional state during shopping	*r* = 0.22 **	<0.01
		Predictors		
		Emotional state during shopping	*β* = 0.17 **	<0.01
		Usage of loyalty cards	*β* = 0.12 *	<0.05
**H2**	Quality of life	Correlates		
		Applications importance	*r* = 0.16 **	<0.01
		Warranty management ease of use	*r* = 0.20 **	<0.01
		Warranty application openness	*r* = 0.15 **	<0.01
		Brands application openness	*r* = 0.22 **	<0.01
		Attitude towards online shopping	*r* = 0.22 **	<0.01
		Emotional state during shopping	*r* = 0.13 *	<0.05
		Predictors		
		Warranty management	*β* = 0.15 *	<0.05
**H3**	Quality of life	Procedural effort & digital services adoption	*β * = −0.84, ΔR^2^ = 0.08, F = 5.46, SE = 0.34 **	<0.01
		Cognitive confusion & digital services adoption	*β * = −0.70, ΔR^2^ = 0.09, F = 6.44, SE = 0.30 **	<0.01
		Procedural effort & adoption of warranty applications	*β * = −0.77, ΔR^2^ = 0.06, F = 3.84, SE = 0.33 **	<0.01
		Cognitive confusion & adoption of warranty applications	*β * = −0.56, ΔR^2^ = 0.07, F = 4.78, SE = 0.28 **	<0.01

Note: * *p* < 0.05, ** *p* < 0.01.

## Data Availability

The datasets used and/or analyzed during the current study are available from the corresponding author upon reasonable request.

## Appendix A

Survey measures used in the present study:

1. Customer Complaining Effort

Procedural effort

It took great time and effort to provide documents required to prove my dissatisfaction.

I was not able to get my problem resolved when I first contacted the company because I was not able to provide required paperwork.

It was not easy to reach the customer service center.

Too much paperwork was required during the process.

Cognitive confusion

I did not know which channel I should use to have my problem resolved.

I did not know who at the company to talk to.

I did not know what to do to have my problem resolved.

I could not find any information about how to file a formal complaint about my experience.

Time-related effort

It took me a long time to have my problem resolved.

I had to contact the company several times to have my problem resolved.

I had to repeat my situation several times to different people at the company.

Having my problem resolved was a difficult process.

Affective effort

I was angry about this experience.

Overall this was a negative experience.

I was happy about this experience.

This was a depressing experience.

Response scale: 5-point Likert scale: never (1)–always (5)

2. Shopping Well-Being (SHWB)

Thinking about shopping, I feel that my shopping contributes significantly to my own personal well-being.

Thinking about shopping, my quality of life would diminish significantly if I don’t shop.

Thinking about shopping, I feel that shopping makes me happy.

Thinking about shopping, I feel that shopping contributes significantly to my quality of life overall.

I feel that my shopping activities contribute significantly to my family well-being.

The quality of life of my family would diminish significantly if I don’t shop.

I feel that shopping makes me happy because shopping contributes much to my family well-being.

I feel that my shopping contributes significantly to my family’s quality of life overall.

Response scale: 7-point Likert scale: strongly disagree (1)–strongly agree (7)

3. Consumer Profile
3.1. Likert items

The consumers’ attitude towards online applications was

What is your attitude regarding the usage of online applications?

Response scale: 6-point Likert scale: not open at all (1)–totally open (6)

The digital services behavior adoption

If a specific brand will develop a new app, how open are you to use it?

Response scale: 5-point Likert scale: not open at all (1)–totally open (6)

The respondents’ digital warranty management adoption

How interested are you to use a new warranty management app instead of the papers received from the shop?

Response scale: 5-point Likert scale: not open at all (1)–totally open (5)

The warranty management easiness

How easy is it for you to manage the warranty of the products?

Response scale: 5-point Likert scale: very difficult (1)–very easy (5)

The emotional state during shopping

What is your emotional state while you shop?

Response scale: 5-point Likert scale: very tired (1)–very relaxed (5)

The behavior regarding the loyalty cards

How much do you use the loyalty cards?

Response scale: 5-point Likert scale: never (1)–always (5)

The applications’ importance

How important is to you for a store to have online shopping apps

Response scale: 5-point Likert scale: not important at all (1)–very important (5)

3.2. Multiple-choice items

The preferred shopping method

Which is your preferred way to shop?

Answers: “physical shops”, “phone orders”, “both online and offline shopping”, “online orders, on the shop’s website” and “online shopping, through shops’ applications”.

Reasons consumers prefer the online shopping experience

Which is the main reason you buy online?

Answers: “time management”, “price”, “comfort”, “to avoid social gatherings”, “larger stock compared to the physical store”. “I do not order online. I like going to the physical store”, “the shopping process is easier and faster” and “other reasons”.

Budget

What is your weekly shopping budget?

Answers: “less than 60 Euros”, “between 60 Euros and 120 Euros”, “between 121 Euros and 180 Euros” and “more than 180 Euros”.

*Prices were displayed in Romanian currency.

Time consumers spent shopping

How much time do you spend shopping per week?”

Answers: “less than 1 h”, “1–2 h”, “between 2 and 5 h”, “more than 6 h”, “I don’t go to physical shops, I buy products online quickly”, “I buy online, but it takes 1–2 h to compare offers and check the products’ specifications” or “I buy online, but it takes 3–4 h to compare offers and check the products ‘specifications”.

Reasons consumers choose the products

Which is the criteria you use while buying the products?”

Answers: “the products advertising”, “your personal opinions about the product”, “the positive experience with the product”, “I have certain favorite brands that I buy regularly”, “the interaction with the brand is simple”, “I buy from brands that have innovative/different products in their category”, “the brand is trustworthy”, “I buy only products that satisfy my needs in a certain moment” and “other reasons”.

Preferred payment method

Which is your preferred payment method?

Answers: “online payment applications”, “credit card”, “I prefer to pay cash” or “I prefer to pay with meal vouchers”.
